# Antibacterial Effect of Prodigiosin on Uropathogenic *Escherichia coli*

**DOI:** 10.1155/sci5/6668394

**Published:** 2025-12-28

**Authors:** Nikhitha Joshy, Biranthabail Dhanashree

**Affiliations:** ^1^ Department of Microbiology, Kasturba Medical College Mangalore, Manipal Academy of Higher Education, Manipal, India, manipal.edu

**Keywords:** biofilm, *E. coli*, *fim*H, prodigiosin, *Serratia marcescens*

## Abstract

Antibiotic resistance and biofilm formation are becoming more common in uropathogenic *Escherichia coli* (UPEC). Hence, the study aims to determine the antibiogram of commonly prescribed antibiotics and assess prodigiosin’s antibacterial activity against UPEC. During the study, 175 UPEC isolates were identified biochemically, and their antibiogram was studied by the VITEK 2 system. Prodigiosin was extracted from *Serratia marcescens* MTCC 97. The MIC of prodigiosin against UPEC strains was detected by the microbroth dilution method. The majority of the UPEC strains (*n* = 135) had MIC between 15 and 30 mg/mL. No significant association was observed between the MIC of prodigiosin and the antibiogram. Biofilm assay was performed by the microtiter plate method using media with and without added prodigiosin. In media without prodigiosin, most UPEC isolates were nonbiofilm formers (NBF—55.42%), followed by weak (21.14%), moderate (MBF—13.71%) and strong biofilm formers (SBF—9.7%). When the same test was performed in media with added prodigiosin, NBF decreased to 30.28%, while SBF, MBF and WBF increased to 20%, 26.85% and 22.85%, respectively. This change in biofilm production was statistically significant (*p* < 0.05, Wilcoxon signed‐rank test). The effect of prodigiosin on *fim*H virulence gene was evaluated using PCR. The *fim*H gene was present in 159 (90.85%) isolates cultured in medium devoid of prodigiosin, whereas in prodigiosin‐containing media, 132 (83.01%) isolates were positive for the *fim*H gene and 27 (16.98%) were negative (*p* < 0.05, McNemar’s test), suggesting that the *fim*H‐negative isolates had either considerable suppression of the gene’s transcription or gene expression pathways. Further, biofilm production increased when prodigiosin inhibited the *fim*H gene, suggesting a gene‐dependent reaction that may have compelled UPEC to adopt a stress–response phenotype that favours nonspecific surface attachment to form biofilm for survival. Therefore, the effect of prodigiosin on biofilms seems to be associated with the expression of *fim*H, indicating a gene‐dependent response.

## 1. Introduction

Uropathogenic *Escherichia coli* (UPEC), *E. coli* responsible for urinary tract infections (UTIs), causes 75% of uncomplicated and over 50% of complicated UTIs [1]. UPEC strains possess extra genetic material found on pathogenicity islands (PAIs), which encode virulence factors, such as haemolysin, iron acquisition systems, lipopolysaccharides and adhesins [2]. The major virulence factors, type 1 fimbriae and the fimbrial protein H (*fim*H), facilitate adherence to the urothelium and help UPEC withstand urine flow, thus helping in colonization, further leading to UTI [3].

A biofilm is a bacterial aggregation in an extracellular matrix of polymeric materials that protects the microorganisms from adverse conditions [4]. Biofilm plays a crucial role particularly in catheter‐associated UTI (CAUTI) [5]. A major challenge in using antibiotics to get rid of biofilms is achieving the necessary minimum inhibitory concentration (MIC) of the drug at the site of infection [6].

One of the most important issues the world is currently experiencing is multidrug resistance (MDR). MDR *E. coli* is described as an *E. coli* strain that, according to *in vitro* susceptibility testing, has become nonsusceptible to at least one compound in three or more antimicrobial classes [7]. According to the *World Health Organization (WHO) Bacterial Priority Pathogens List 2024*, antibiotic‐resistant *E. coli* has been classified among the high‐priority pathogens due to its increasing resistance to commonly used antibiotics including third‐generation cephalosporins, carbapenems and its significant contribution to UTI worldwide. The report emphasizes the urgent need for developing new antimicrobial agents and alternative therapeutic strategies to combat infections caused by MDR *E. coli* [8]. In this context, studying the current drug susceptibility pattern and investigating novel bioactive compounds, such as prodigiosin against UPEC, are highly relevant.

As the antibiotic resistance in UPEC is on the rise due to either transfer of drug resistance or biofilm formation in catheters, we studied the susceptibility of commonly used antibiotics and explored the antibacterial effect of the naturally available compound prodigiosin on UPEC as an alternative antibacterial agent.

## 2. Materials and Methods

### 2.1. Study Design and Sampling Method

This cross‐sectional, time‐bound study collected 175 clinically significant *E. coli* isolates from urine samples received at the Department of Microbiology, Kasturba Medical College, Mangalore, using the convenient nonrandom sampling method. *E. coli* was considered clinically significant if recovered from urine samples having pus cells with a colony count greater than 10^3^ CFU/mL. Unless explicitly stated, all the study’s chemicals, media, antibiotic discs and ATCC reference strains were purchased from HiMedia Laboratories Pvt Ltd., Mumbai, India. The Institutional Ethical Committee (IEC) granted permission for the conduct of this research [IEC KMC MLR 05/2024/309].

### 2.2. Bacterial Strains Used in the Study

*S. marcescens* MTCC 97 is known for its ability to produce bright red pigment called prodigiosin. Hence, we used this strain for the production of prodigiosin. This strain was procured from Microbial Type Culture Collection and Gene Bank, Chandīgarh, India. The use of this bacterial strain ensured consistency and reliability in pigment yield and purity.

*E. coli* ATCC 25922 was primarily used as a reference strain for antibiotic susceptibility testing (AST) and quality control. It has smooth lipopolysaccharide, specific porin profile and common adherence genes (such as *fim* and *pga* operon), and possesses prophage elements, with a 5.2 Mb genome containing genes for metal resistance. We used this reference strain for determining the MIC of prodigiosin and as a positive control in PCR to detect the presence of *fim* H gene.

*Pseudomonas aeruginosa* ATCC 27853 is known for its strong biofilm‐forming ability and unique gene expression, especially concerning adhesion and virulence factors, such as pili and flagella, making it ideal for AST and biofilm research. We used *P. aeruginosa* ATCC 27853 as a positive control in the biofilm assay as this strain is a strong biofilm producer.

*E. coli* ATCC 25922 and *P. aeruginosa* ATCC 27853 were procured from HiMedia Laboratories Pvt Ltd., Mumbai, India. All these strains were maintained on nutrient agar slants at 4°C and subcultured periodically to maintain the viability and characteristics of these strains.

### 2.3. Identification, Antibiotic Sensitivity Testing and Preservation of Isolates

Clinically relevant UPEC strains were identified in the microbiology laboratory by biochemical reaction, and AST for routinely used antibiotics was performed by using the VITEK 2 system (BioMérieux, USA). The antibiotics tested included ampicillin, amoxicillin/clavulanic acid, ticarcillin, piperacillin–tazobactam, cefixime, ceftazidime, ertapenem, amikacin, gentamicin, nalidixic acid, ciprofloxacin, ofloxacin, fosfomycin, nitrofurantoin, cotrimoxazole, norfloxacin, meropenem, imipenem, netilmicin, cefoperazone–sulbactam and ceftriaxone. *E. coli* ATCC 25923 is used as a quality control strain in the AST, and results were interpreted as per the CLSI guidelines [9]. The UPEC isolates were preserved in 20% glycerol broth at −80°C.

### 2.4. Preparation of Prodigiosin Extract

A few colonies of *Serratia marcescens* MTCC 97 were inoculated in 300 mL of peptone water (pH 7.2) and incubated for 5 days at 27^o^C. The culture was centrifuged at 7500 rpm for 20 min, and the bacterial pellets were treated with a 3:1 mixture of acetone and methanol until colourless and centrifuged at 7500 rpm for 5 min. After passing through Whatman No. 1 filter paper, the coloured supernatant was transferred to a sterile glass Petri plate and allowed to dry overnight at 37°C in an incubator. This dry pigment was dissolved at a concentration of 100 mg/mL in 99.8% methanol [10].

### 2.5. Biophysical Characterization of Prodigiosin

Using Vanquish liquid chromatography in conjunction with Thermo Fisher’s Orbitrap (Q Exactive) mass spectrometry, the quality and potential components of the prodigiosin crude extract were evaluated, at Manipal School of Life Sciences, Manipal, India. Methanol and chloroform (1:1) were utilized as the mobile phase, while capillary columns fused with silica gel (250 × 4.6 mm) served as the stationary phase. Water with 0.1% formic acid (solvent A) and methanol with 0.1% formic acid (solvent B) were employed as a gradient elution solvent. To separate pigments according to their hydrophobicity, the gradient gradually moved from a high percentage of aqueous solvent to a high percentage of organic solvent. 4‐Methoxy‐2,2′‐bipyrrole‐5‐carbaldehyde (MBC) and 2‐methyl‐3‐n‐amylpyrrole (MAP) were the precursor ions chosen for investigation based on the mass‐to‐charge (m/z) ratios of protonated or deprotonated molecular ions of prodigiosin [11].

To generate distinctive fragment ions in higher‐energy collisional dissociation (HCD) or collision‐induced dissociation (CID) modes, collision energies were varied. The stability and fragmentation pattern of prodigiosin were taken into consideration when adjusting typical collision energy. Following processing of the MS data, the LC/MS fragmentation and molecular profiles were compared with reference spectra of recognized bacterial pigments found in commercial spectral libraries and databases. The molecular weight of prodigiosin was calculated, the fragment ions were predicted, and the identity of prodigiosin was confirmed using Thermo Fisher’s Mass Frontier software Version 8.1. The METLIN database was utilized for bioinformatics and statistical research.

### 2.6. Detection of MIC of Prodigiosin for UPEC

Two to three isolated colonies of UPEC strains and *E. coli* ATCC 25922 were suspended in 3 mL of cation‐adjusted Muller–Hinton broth (CAMHB) to create the standard inoculum. To achieve a concentration of 5 × 10^6^ CFU/mL, the inoculum turbidity was corrected to 0.5 McFarland standard and then further diluted 1:20 with sterile saline. Each well of the round‐bottom microtiter plate (MTP) received 100 μL of CAMHB. Prodigiosin was pipetted to the wells of the MTP in varying concentrations (5, 10, 15, 20, 25 and 30 mg/mL). Ten microlitres of the 1:20 diluted bacterial inoculum was added to each well. *E. coli* ATCC 25922 was used as a positive control. The sterility control used was CAMHB with prodigiosin and no bacterial inoculum. Growth control used was CAMHB without prodigiosin injected with 10 μL of *E. coli* ATCC 25922. The MTPs were incubated for 24 h at 37°C. The turbidity and colour of the test wells were compared to those of the growth control and sterility control wells to visually read the MIC. The microtiter well with the lowest prodigiosin concentration that prevented the growth of UPEC was referred to as the MIC [9, 12]. One concentration below the MIC was taken as subinhibitory concentration (SIC). Using the reference strain of *E. coli* ATCC 25922, the inhibitory effects of solvents, such as acetone and methanol, which are used to extract and dissolve the pigments, were examined in CAMHB plates at concentrations ranging from 10% to 50%. These concentrations of solvents did not inhibit the growth of reference strains of *E. coli*.

### 2.7. Detection of the Effect of Prodigiosin on Biofilm by the MTP Method

The ability of UPEC to produce biofilms was assessed using a MTP test, both with and without prodigiosin. The biofilm experiment was initially carried out on all 175 UPEC isolates and reference strain using Luria–Bertani (LB) broth without prodigiosin. One millilitre of LB broth was inoculated with one loopful of the respective UPEC isolates, and the broth was incubated for 18 h at 37°C. To achieve a turbidity of 0.5 McFarland standard, these cultures were diluted (1:20) with sterile saline. Sterile 96‐well polystyrene round‐bottom, MTPs with lids (Labtech Medico Pvt Ltd., Kerala, India) were filled with 100 μL of the diluted UPEC culture in triplicate. As a positive control, strong biofilm‐forming *P. aeruginosa* ATCC 27853 was utilized. LB broth without bacterial inoculum was used as a negative control. Plates were incubated at 37°C for 48 h aerobically. Wells of the plates were washed thrice with 200 μL of 1 × phosphate buffer saline (PBS). About 200 μL of 99% methanol was introduced to the wells and left at room temperature for 15 min. Methanol was removed, and MTPs were dried for 15 min. For staining, 200 μL of 0.5% crystal violet solution was dispensed in the wells of MTP and was kept at room temperature for 5 min. Stain was washed using distilled water. After drying, 150 μL of 95% ethanol was used for dissolving the stain bound to biofilm. Optical density was measured at 570 nm in a MTP reader (Multiskan FC Filter‐based Microplate Photometer, Thermo Fisher Scientific) [13]. Experiments were repeated three times. The experiment was replicated as described above using LB broth supplemented with subinhibitory quantities of prodigiosin to determine its impact on biofilm. Three standard deviations (SDs) over the negative control mean were taken as the cutoff (ODc). Biofilm of the isolates was classified based on mean optical density of isolate (ODi) relative to ODc: nonbiofilm formers (NBF = [ODi < ODc]), weak biofilm formers (WBF = [ODc ≤ ODi ≤ 2ODc]), moderate biofilm formers (MBF [2ODc < ODi ≤ 4ODc]) and strong biofilm formers (SBF [ODi > 4ODc]) [14].

### 2.8. Detection of *fim*H Virulence Gene by Polymerase Chain Reaction

DNA extraction from the UPEC was performed using the boiling method [15]. Extracted DNA was stored at −20°C. Nuclease‐free water lacking DNA served as nontemplate/negative control, while DNA of *E. coli* ATCC 25922 was employed as the positive control. Primers used in the PCR are shown in Table 1.

**Table 1:** Primers used for PCR.

Primer	Sequence	Product size (bp)	Reference
Forward	5′‐TGC​AGA​ACG​GAT​AAG​CCG​TGG‐3′	506	Bali et al. [16]
Reverse	5′‐GCA​GTC​ACC​TGC​CCT​CCG​GTA‐3′		

PCR was performed with 12.5 μL ready‐to‐use master mix (DSS Takara Bio India Pvt. Ltd.), 2.5 μL template, 1 μL of both primers (0.4 μM) and 8 μL nuclease‐free water, for a total volume of 25 μL. Thermal cycling (Thermo Fisher Scientific—IN) for *fimH* involved initial denaturation at 95°C for 5 min, followed by 35 cycles: denaturation at 94°C for 30 s, annealing at 55°C for 30 s, extension at 72°C for 1 min and final extension at 72°C for 10 min. PCR products were analysed by electrophoresis on 1.5% agarose gel and captured using a documentation system (Bio‐Rad GEL DOC GO) [16].

### 2.9. Impact of Prodigiosin on *fim*H Virulence Gene

To know the impact of prodigiosin on *fim*H gene, UPEC isolates were grown in LB broth containing SICs of prodigiosin for 18 h at 37°C. This broth was centrifuged at 7000 rpm for 10 min. DNA was extracted from the pellet by the boiling method. PCR was performed on these prodigiosin‐treated isolates using the same primer and cyclic condition as mentioned earlier [16].

### 2.10. Data Analysis

The experiments were conducted three times in triplicate. The main goal of these technical and biological replicates is to lessen any variances in the experimental findings. The test value mean ± SD is used to display all results. The results were tabulated in Microsoft Excel, and JAMOVI software (Version 2.6.26) for Windows was used to analyse the results. The association between the MIC of prodigiosin and the resistance profile of commonly used antibiotics was determined using the Mann–Whitney *U* test. The difference between the formation of biofilms with and without prodigiosin was assessed using the Wilcoxon rank sum test. The McNemar test was employed to determine whether the presence or lack of prodigiosin was associated with any statistically significant differences in the presence or absence of the *fim*H gene. A *p* value of less than 0.05 was deemed statistically significant.

## 3. Results

### 3.1. Characteristics of Studied UPEC Isolates

Of the 175 UPEC isolates, 111 (63%) came from females and 64 (37%) from males, indicating a female preponderance. The prevalence of UPEC infection is higher among elderly patients of age group 60–90 years (75; 42.9%), followed by middle‐aged in the group of 36–59 years (64; 36.6%), young adults in the age group 18–35 years (23; 13.1%) and children aged below 17 years (13; 7.4%). The prevalence of UPEC infections and MDR in different age groups is depicted in Table 2. UPEC showed high resistance to nalidixic acid (86.28%), ampicillin (79.43%) and ticarcillin (78.86%), while remaining highly susceptible to ertapenem (98.85%), fosfomycin (98.28%) and nitrofurantoin (80.57%) (Table 3). ESBL production was observed in 41.71% of isolates. Carbapenem resistance was seen in 19%, and MDR was observed in 82% of isolates (Table 2).

**Table 2:** Prevalence of uropathogenic *E. coli* (UPEC) and multidrug resistance in different age groups.

Age group in years	Number of isolates by patient sex			Number of multidrug‐resistantUPEC	Number of ESBL‐producing UPEC	Number of carbapenemase‐producingUPEC
	Male	Female	Total			
< 1–10	4	6	10	8	4	1
11–20	2	2	4	3	1	1
21–30	5	14	19	13	6	2
31–40	3	9	12	9	3	2
41–50	6	13	19	18	9	4
51–60	18	23	41	34	19	11
61–70	14	17	31	26	14	5
71–80	10	19	29	26	14	8
81–90	2	8	10	6	3	0
Total	64	111	175	143	73	34

**Table 3:** Susceptibility of studied UPEC isolates to screened antibiotics.

Antibiotics	Sensitive *N* (%)	Intermediate *N* (%)	Resistant *N* (%)
Ampicillin	35 (20)	1 (0.57)	139 (79.43)
Amoxicillin/clavulanic acid	89 (50.85)	26 (14.85)	60 (34.28)
Ticarcillin	36 (20.57)	1 (0.57)	138 (78.86)
Piperacillin–tazobactam	120 (68.57)	0	55 (31.43)
Cefixime	52 (29.71)	1 (0.57)	122 (69.71)
Ceftazidime	93 (53.14)	2 (1.1)	80 (45.71)
Ertapenem	173 (98.85)	0	2 (1.14)
Amikacin	136 (77.71)	10 (5.71)	29 (16.57)
Gentamicin	114 (65.14)	2 (1.14)	59 (33.71)
Nalidixic acid	24 (13.71)	0	151 (86.28)
Ciprofloxacin	42 (24)	5 (2.8)	127 (72.57)
Ofloxacin	57 (32.57)	2 (1.14)	116 (66.28)
Fosfomycin	172 (98.28)	0	3 (1.7)
Nitrofurantoin	141 (80.57)	17 (9.7)	17 (9.7)
Cotrimoxazole	91 (52)	0	84 (48)
Norfloxacin	62 (35.42)	4 (2.28)	109 (62.28)
Meropenem	141 (80.57)	0	34 (19.43)
Imipenem	139 (79.42)	2 (1.14)	34 (19.42)
Netillin	149 (85.14)	0	26 (14.86)
Cefoperazone–sulbactam	138 (78.85)	2 (1.14)	35 (20)
Ceftriaxone	60 (34.28)	1 (0.57)	114 (65.14)

### 3.2. Extraction of Pigment

**Figure 1:**
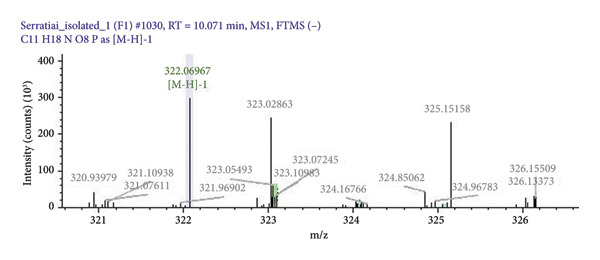
Spectrograph of prodigiosin generated by LC/MS. Peak seen in green colour represents the molecular weight (323.10) of prodigiosin.

*Serratia marcescens* MTCC 97 when grown in 300 mL of peptone water and extracted with acetone and methanol yielded 150 mg of dry pigment. This red pigment produced was characterized by LC/MS to confirm the compound and molecular structure. The antimicrobial compound in the red pigment of *Serratia marcescens* MTCC 97 was confirmed as prodigiosin with a molecular weight of 323 g/mol as shown in Figure 1.

### 3.3. MIC of Prodigiosin for UPEC

**Figure 2:**
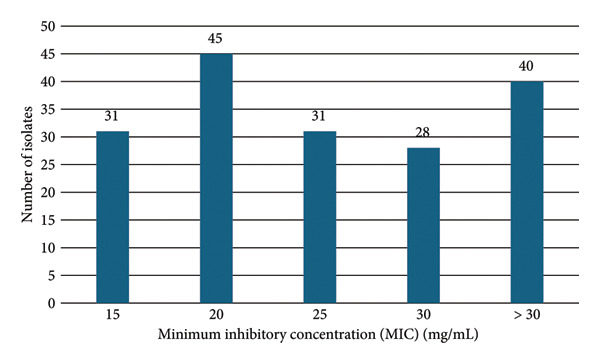
Prodigiosin MIC for studied UPEC isolates (*n* = 175) as determined by the microbroth dilution method.

MIC of prodigiosin was detected by the MTP method for all the 175 UPEC isolates. Prodigiosin showed strong inhibitory activity against UPEC, with MIC values ranging from 15 to 30 mg/mL as shown in Figure 2. The MIC for *E. coli* ATCC 25922 was 15 mg/mL. Among the urinary isolates, 22.85% had MIC > 30 mg/mL, while others had MICs of 30 mg/mL (16%), 25 mg/mL (18.28%), 20 mg/mL (25.71%) and 15 mg/mL (17.14%). No significant association was found between prodigiosin MIC and resistance or susceptibility to routinely used antibiotic (*p* > 0.05, Mann–Whitney *U* test).

### 3.4. Effect of Prodigiosin on Biofilm Formation by the MTP Method

**Figure 3:**
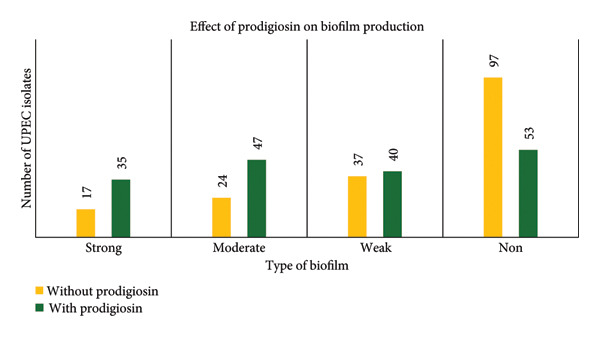
Comparison of biofilm production by UPEC isolates using medium with and without prodigiosin by the microtiter plate method.

Biofilm‐forming ability of the UPEC isolates was studied using LB broth without prodigiosin and with SICs of prodigiosin using the MTP method. In LB broth without prodigiosin, most UPEC isolates were NBF (55.42%), followed by weak (21.14%), MBF (13.71%) and SBF (9.7%). When grown in LB broth containing SICs of prodigiosin, NBF decreased to 30.28%, while SBF, MBF and WBF increased to 20%, 26.85% and 22.85%, respectively. This change was statistically significant (*p* < 0.05, Wilcoxon signed‐rank test). Comparison of biofilm production in the presence and absence of prodigiosin is depicted in Figure 3.

### 3.5. Impact of Prodigiosin on *fim*H Gene

**Figure 4:**
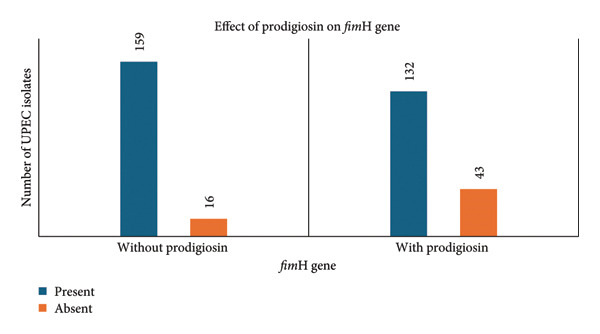
Detection of *fim*H gene by PCR after growing UPEC isolates in medium with and without prodigiosin.

*fim*H gene was detected by PCR in the UPEC isolates after growing the isolates in LB broth with and without SICs of prodigiosin. Among the 175 UPEC isolates that were grown in LB broth without prodigiosin, 159 (90.85%) had the *fim*H gene. Following cultivation of UPEC isolates in LB broth supplemented with a SIC of prodigiosin, 132 (83.01%) isolates were positive and 27 (16.98%) were negative for the *fim*H gene, suggesting inhibition of either gene expression pathways and type 1 fimbriae assembly or transcription of the *fim*H gene into mRNA in 27 *fim*H‐negative UPEC isolates. The McNemar test showed that prodigiosin has a statistically significant impact on the *fim*H gene (*p* < 0.05). The effect of prodigiosin on *fim*H as detected by PCR is shown in Figure 4.

The association between biofilm and *fim*H gene in the presence and absence of prodigiosin was calculated by the Wilcoxon rank sum test. The *fim*H gene is associated with higher biofilm formation, and this effect is amplified by prodigiosin, whereas in the absence of *fim*H, prodigiosin does not significantly impact biofilm formation (*p* value < 0.05).

## 4. Discussion

*E. coli* is the major etiological agent of UTI, which adheres to uroepithelial cells and leads to biofilm formation. Biofilm increases resistance to commonly used antibiotics which has necessitated the development of novel antimicrobial approaches, such as bacterial pigments [2]. This study demonstrates the susceptibility pattern of UPEC to commonly used antibiotics and evaluates antimicrobial efficacy of prodigiosin on UPEC, exploring its potential as a novel therapeutic option.

In this study, UPEC infections were more prevalent (63.4%) among females (Table 2), likely due to physiological factors, and were consistent with findings from a study in Haryana [17]. Antibiotic resistance in UPEC has been rising rapidly, complicating treatment outcomes worldwide [7, 8, 16–19]. In this study, high resistance was observed against most of the antibiotics tested except for fosfomycin (1.7%) and ertapenem (1.1%) as shown in Table 3. Similar resistance patterns have been reported in studies from Mangalore [19], northeastern Karnataka [20] and in a multicentric Indian studies [18, 21]. A Polish study also noted that resistance to amoxicillin/clavulanic acid, ciprofloxacin and cotrimoxazole is more pronounced in developing countries [22].

Primary resistance mechanisms in UPEC are the production of extended‐spectrum β‐lactamases (ESBL) and carbapenemases which lead to the emergence of MDR strains with limited treatment options [7, 8]. An earlier Indian study reports 34% ESBL‐producing and 25% carbapenemase‐producing UPEC strains among the UPECs collected from all geographical regions of India [18]. A study from Lebanon reports 64% ESBL producers and 68% MDR strains and no carbapenem‐producing UPEC strains [23]. Compared to previous Indian research, the examined UPEC isolates had fewer carbapenemase producers (34; 19%), more ESBL producers (73; 41.71%) and MDR (143; 82%) as shown in Table 3. Therefore, it is evident from the findings that antibiotic susceptibility varies by region due to socioeconomic factors, healthcare facilities and antibiotic prescribing policies.

Antimicrobial red compound of *S. marcescens* MTCC 97 was confirmed as prodigiosin (mol.wt323 g/mol) based on the m/z ratio [24]. The molecular weight of prodigiosin that was used in MIC study is shown in Figure 1. Prodigiosin has shown antimicrobial properties against various microorganisms in different studies [13, 24]. In this study, prodigiosin showed significant inhibitory effect on UPEC. As seen in Figure 2, approximately 77.14% of UPEC had prodigiosin MICs between 15 and 30 mg/mL, with the remainder having MICs greater than 30 mg/mL. No statistically significant association was seen between MIC of prodigiosin and the susceptibility pattern of routinely used antibiotics (*p* > 0.05). A study conducted in Malaysia used prodigiosin against reference strains of various bacteria including *E. coli* and MIC of prodigiosin for *E. coli* was 10 mg/mL [13]. In our study, the MIC of prodigiosin for *E. coli* ATCC 25922 was 15 mg/mL. From these findings, we can conclude that prodigiosin MIC or susceptibility also varies by geographic location.

Chronic and recurring UTIs are linked to biofilm‐forming UPEC. Biofilms help UPECs survive in the urinary tract by protecting the bacteria from drugs and the host immune system [25]. However, because 55.42% of the isolates in this investigation were NBF, in LB broth without prodigiosin the study concluded that biofilm formation was not a prominent mechanism in UPEC isolates from this part of the world. Only 13.5% of UPEC isolates developed biofilm, according to a study from Haryana [17], but 54.1% of isolates produced biofilm in a study conducted in Kathmandu [26]. However, a study from Uganda reports 62.5% of their UPEC strains to be biofilm formers [27]. Different genetic compositions, quorum sensing, variations in extracellular polymeric substances and environmental factors may contribute to the diversity in biofilm development across UPEC isolates from various geographic locations.

As depicted in Figure 3, when UPEC were grown in the presence of SIC of prodigiosin, the number of biofilm formers increased and NBF decreased. This prodigiosin‐enhanced biofilm formation is possibly due to stress responses, membrane disruption or quorum sensing, and warrants further investigation. In contrast, a Malaysian study observed a slight reduction in biofilm formation in a single reference *E. coli* OP50 strain treated with prodigiosin [13]. Earlier studies have tested prodigiosin’s effect only on few clinical or reference strains [12, 28]. To the best of our knowledge, this is the first study to show the effect of prodigiosin on good number of UPEC strains, its virulence gene and biofilm. Statistical comparison of biofilm production in the presence and absence of prodigiosin using the Wilcoxon rank sum test showed the results to be statistically significant (*p* < 0.001; *p* value < 0.05 is significant). Dawadi et al. have shown that hydrogen peroxide enhances the biofilm‐forming ability among nonproducers, weak and moderate biofilm producers despite the presence or absence virulence genes [29], but the limited data on prodigiosin’s antibiofilm activity on UPEC highlight the need for more research.

The *fim*H gene of UPEC encodes a subunit of type 1 fimbriae, which is essential for adhesion and biofilm formation and is present in 68%–96% of UPEC [30]. In this study, the *fim*H gene was not found in 27 of the 159 *fim*H‐positive UPEC isolates cultivated in LB broth with SIC of prodigiosin. This suggests that either gene expression pathways and type 1 fimbriae assembly or transcription of the *fim*H gene into mRNA was inhibited, which requires further research. The effect of SIC of prodigiosin on *fim*H gene is statistically significant (*p* value < 0.001; McNemar’s test; *p* value < 0.05 is significant). No prior data exist on prodigiosin’s effect on the *fim*H gene. Biofilm production increased when prodigiosin inhibited the *fim*H gene, suggesting a complicated, gene‐dependent reaction that may have compelled UPEC to adopt a stress–response phenotype that favours nonspecific surface attachment in order to form biofilm for survival. This highlights the need for further studies to clarify prodigiosin’s mechanism on virulence gene regulation.

Despite the fact that this work assesses prodigiosin’s impact on several UPEC isolates and their virulence factors in a novel way, there are still some limitations. Prodigiosin’s mode of action is yet unclear, and using pure pigment could result in more potent antibacterial activity. However, its clinical use is limited by its low bioavailability, restricted spectrum of action, instability and challenges in large‐scale production. Further animal studies and clinical trials are needed to confirm its efficacy and safety as a potential therapeutic agent against UPEC infections.

## 5. Conclusions

This study assessed prodigiosin’s antibacterial activity against UPEC, including its MIC, impact on biofilm formation and on *fimH* gene. While prodigiosin showed antimicrobial activity, its role in biofilm enhancement and gene expression warrants further investigation. Understanding its dual effects is key to evaluating its potential as a therapeutic agent.

## Ethics Statement

The Institutional Ethics Committee (IEC KMC MLR 05/2024/309) of Kasturba Medical College, Mangalore, has approved this study.

## Disclosure

All authors read and approved the final version of the manuscript.

## Conflicts of Interest

The authors declare no conflicts of interest.

## Author Contributions

N.J.: data collection, conduct of experimental work, formal analysis of data and interpretation and drafting of the manuscript.

B.D.: conceptualization and designing of methodology, editing and rewriting of manuscript and supervision of the entire work.

## Funding

This research did not receive any specific funding.

## Data Availability

The datasets used and/or analysed during this study are available from the corresponding author upon reasonable request.
